# Design and Evaluation of Electronic Briefs of Neonatal Intensive Care Unit in Taleghani Hospital, Tabriz, Iran

**DOI:** 10.5539/gjhs.v6n5p125

**Published:** 2014-05-16

**Authors:** Kayvan Mirnia, Taha Samad Soltani, Manouchehr Rezaei, Mohammad Heidarzadeh, Zakieh Piri

**Affiliations:** 1 Pediatric Health Research Center, Tabriz University of Medical Sciences, Tabriz, Iran; 2 Department of Medical Informatics, Paramedicine Faculty, Tehran University of Medical Sciences, Tehran, Iran; 3 Department of Health Information Technology (HIT), Faculty of Medical Library and Information Science, Tabriz University of Medical Sciences, Tabriz, Iran

**Keywords:** database, neonatal, nicu, electronic medical record

## Abstract

More than 9 million neonatal deaths are reported through out the world each year happening in the early weeks of life most of which relate to developing countries. Thus it is very important to present a better way to keep the infants healthy which could be possible by accessing accurate information at any time required during hospitalization of infants. Therefore the required data should be collected, stored and analyzed before which is best possible by using computer. The main objective of this research is enabling researchers and clinicians quick access to the data of the babies admitted in NICU. This study involves the stage of developing a system design and its implementation following the evaluation of the electronic records which is done in a query form. By defining the neccessar terminology and designing a data model, the database and user interface are developed by using a programing language and data base tools. Finaly, the system has been evaluated by user satisfaction showing to be about 85% As a result we suggest the hospitals take serious in buying the suitable technology for the NICU ward along with teaching the staffs how to work with it.

## 1. Introduction

### 1.1 Introduce the Problem

In reference to Herbst and Drenth (2012), Rachatapantanakorn, Tongkumchum, and McNeil (2009), a report from the World Health Organization shows that more than 9 million neonatal deaths occur worldwide each year in the early weeks of life most of which are occuring in developing countries. So it is important to keep the infants as a vulenerable group in developing countries healthy by presenting better ways of taking care of them (Amani, Barak, Amini, & Dehghan Mohammad, 2006). However, infant mortality is considered to be as an indicator of socio-economic development and thus most of the countries invest highly on improving NICU to save babies with high risk criteria (Kliegman, Behrman, Jenson, & Stanton, 2012). To reduce mortality in infants, it is necessary to take into account the factors such as maternality and those threatening infants as well as using new suitable technologies (Sharifzadeh, Namakin, & Mehrjoofard, 2008).

### 1.2 Explore Importance of the Problem

To provide some appropriate policies to improve neonatal care, accessibility of accurate required information of the infant is important when he/she is hospetilized (Ntuli, Malangu, & Alberts, 2013). Doctors and health care providers in this field face with many difficulties which can be overcome by some information being obtained by some data that should be collected, stored and analyzed (Schulman, 2008). According to the inappropriate status of information and communication technology in Iran and lack of reliable infrastructure, the data are recorded on the paper and then transferred to the archives leading to numerous problems including illegibility, lack of concentration and time consuming on data mining or even loss of some events. Transferring experiences based on the paper records will face numerous problems, including illegibility, lack of concentration and time consuming on data mining or even loss of some events (Eddy, 2005; Rahmanpour, 2009).

### 1.3 Describe Relevant Scholarship

In a study conducted by Josef schulman (2006) at the hospital in New York City showed that using a centralized database will improve the quality of care and safety of infants in NICU ward and improves the efficiency of the services sector (Schulman, 2008). The importance of this point has been reported by Chido et al. showing that more than 5.5 percent of administration and practice in the NICU suffered medical errors threatening the health of newborns (Kohn, Corrigan, & Donaldson, 2000). Despite the necessity to maintain medical records, most health care institutions are willing to the computerized clinical records (Shortliffe, 1999). Computerized data of the patient are necessary for further researches in this ward. Therefore, fast accessibility to accurate and analyzable data would be helpful in several studies (Horbar, Soll, & Edwards, 2010). Studies show that a detailed and comprehensive database is essential in NICU so that the hospitals can collect adequate and accurate data to be stored in the database which can be helpful in medical interventions and analyzing the relations (Saeed, Lieu, Raber, & Mark, 2002; Sprague et al., 2013).

### 1.4 State Hypotheses and Their Correspondence to Research Design

The main objective of this research is to provide researchers and clinicians quick access to the data of the babies admitted in NICU. For this problem the program of database has been used which showed actually how ever increasing data records of infants can be analyzed resulting in improving the care and prevention errors in contrast to traditional paper based system. Electronic records system has been also evaluated from the user point of view.

The paper is organized as follows: Section 2 invoves the structur of the method used; Section 3 contains the results and the way of the implementation and discussion is the final Section, 4.

## 2. Method

After designing an electronic record in the neonatal ICU, a case study was used (Orlikowski & Baroudi, 1991; Palvia et al., 2004). Case study is a kind of an empirical investigation to search for a new phenomenon in its real state, especially if there is no obvious evidence showing the relation between observasion and its general case (Yin, 1998). The research methods for case study in the information system are well established (Benbasat, Goldstein, & Mead, 1987). A wide variety of methods including qualitative or quantitative one can also be used (Cavaye, 1996). Two methods developing and quantitative are applied here. The system design and implementation have been considered for the first one but evaluation of electronic records has been considered for the second case by asking some questions to be answered by the user through a given form. These two methods are described as follows:

System architecture and some statiscal software as a tool are used to develop a conceptual framework containing the information about the effectiveness of care: (MDS) is the minimum set of data about care unit which is considered to be a base in achieving indicators of effectiveness. Applying standard elements of information and determining required clear information for patient care manager will lead a suitable framework.

Creating a useful database, a set of acceptable terms and necessary characteristics of general situations should be defined clearly for recording the information of the patient in the NICU (Escobar et al., 1997). The set of data involving demographic and clinical information about the infant hospitalized in this unit should be collected.

Data model: designing database tables and their relationships named the model of data is considered to be the core of any electronic records system. The strength of the model of data is being scalable and reflexive systems. In the multi-functional data model systems, the data model is used for several major objectives including: 1) Supporting various functions in the areas of clinical care, facilitate reporting, monitoring, research, and supporting. 2) New data such as medicines, clinical status and outcomes can be added without any change in the data model, since the requirements are not often known completely at the beginning. 3) Provides the opportunity to work with time data. 4) Support multilingual medical terminology. Many electronic medical record systems in developing countries use management databases of relational business systems such as Microsoft Access and SQL server and Oracle or some variants like open sources (Fraser et al., 2005).

User Interface: The user interface method and object-oriented architecture relating user to a database system is relational (S. B. Shneiderman & Plaisant, 2005). Graphics interface is an improved interface for storing and retrieval systems of Information to retrieve from various sources and storing them in the database (Kirk & Levy, 1998).

Database management systems including access provides tools to create a graphical user interface that facilitates using the database. This graphical user interface is made from a set of forms that facilitate well to access the stored data in the table of database or generated by queries (C). Software evaluation: there are many possible ways to evaluate the interface between humans and computers. Schniderman listed five different criteria. For many tasks, speed and accuracy of the performance of the components are related to each other so that it effects on the view of the people to the system. User acceptance of a system is an important component of the success of a system (Shneiderman, 1982). A large number of subjective satisfaction questionnaires of user of the system and many topics corresponding to it have been created, although some of them were just about the evaluation of the user interface.

One of the standard and successful questionnaires to assess user satisfaction with respect to the user interface is the questionnaire that has been presented by China and his colleagues. This questionnaire has a high degree of reliability, high stability and showed to be highly stable. A version of this questionnaire is available by which contains 30 questions about the overall reaction to the software, display unit, terminology and system information, training and system capabilities (Chin, Diehl, & Norman, 1988). To investigate the results of the statistical analysis of the questionnaires, the t-test statistical analysis is used which is applicable for the independent samples and can report the results too.

As mentioned above the least data set has been determined to make table and the fields of information in the data base at the beginning and then the data model has designed for efficient storing and retrieving the information along with designing a user interface in a questionary form. Finally, it has been applied for Taleghani Hospital of Tabriz for 6 months. Then after, the neonatal intensive care information system and also user satisfaction had been evaluated which lead the following results :

## 3. Results

Based on the database in the Vermont oxford, Canadian neonatal network, Schulman and the summarized records available in Alzahra Hospital of Tabriz, neonatal minimum data set has been designed. The created MDS actualy includes the standard variables for storing the data of the patient. The main characteristics of each data such as name, type of the variable (Format) and feature are given. This set is the minimum information index in a standardized format for collecting, storing and transferring neonatal data. This is infact a check list for the patient’s care containing:

1-Demographic data of resuscitation, 2-Maternal risk factors, 3-Information relating to recusitation, 4-Reason of hospitalization, 5-Action during hospitalization either administrating some drugs or helping patients with respiratory devices, 6-Complications during hospitalization, 7-Information at discharge 8-The final diagnosis according to ICD-10 coding system. This form is added to the patients chart and any event is checked by a doctor and nurses every day.

### 3.1 ERD

Data model defined for storing and retrieving the data of newborns is a relational type and the logical relationships between tables and the charts of the entity is shown by ERD. To design the data model, 15 entities have been defined. Any entity in this model bears in fact some parts of baby care information and the patient information plays role of a bridge.

**Figure 1:**
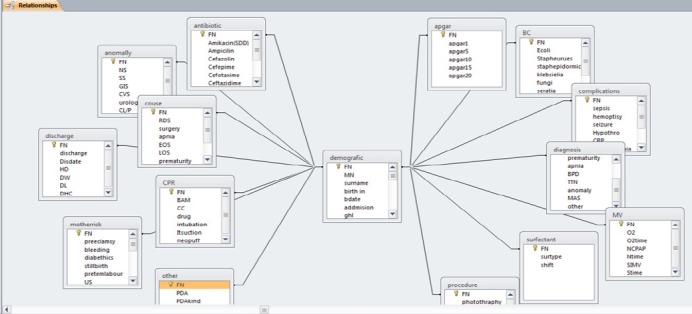
Entities and their relations in developed data model

There is a one to one relation between the table and the central table. Corresponding to each entity in the data model there is a table for keeping the patient data in the database management system. One table is for keeping the demographic information, thirteen tables are for storing the clinical data and one table is for keeping the discharge information. The structure of the data model in infant database is stellar (Figure 1) with the demographic table in the center and the other tables are placed around of it”. The number of the file of the patient” is defined to be the primary key, and the others are connected to each other by using this key.

### 3.2 User Interface of Electronic File System

**Figure 2:**
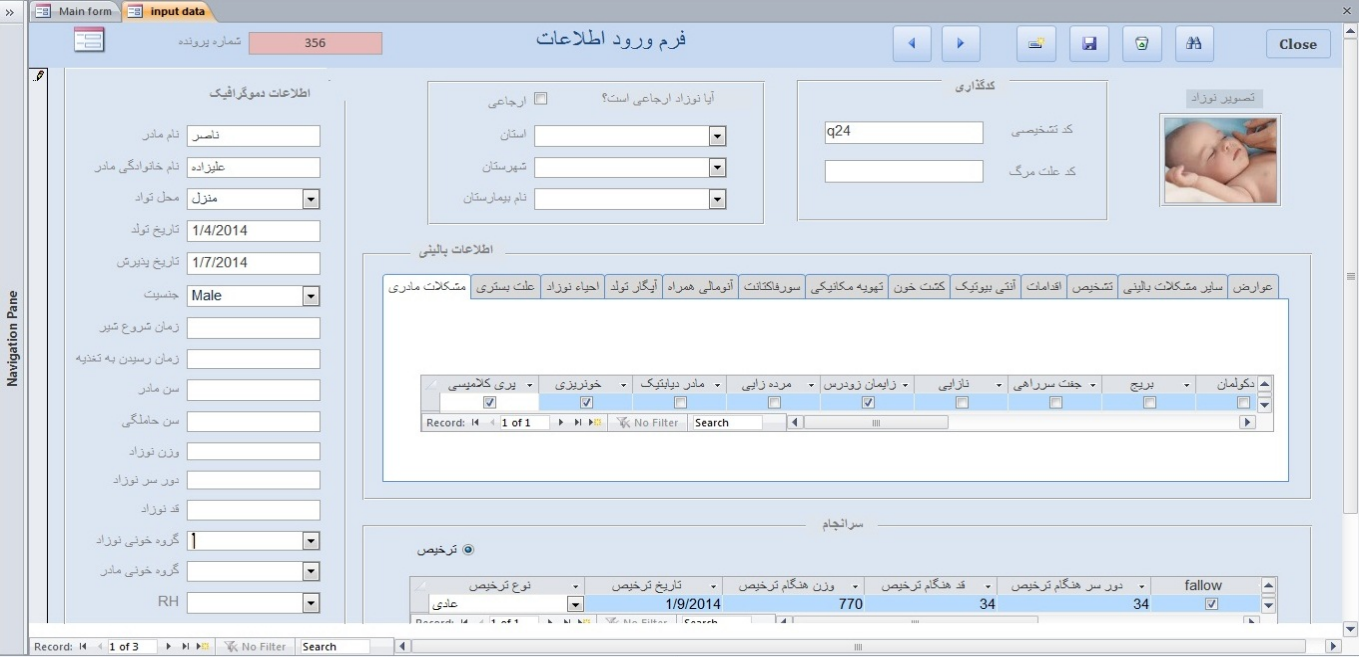
Form of neonatal information

Electronic summary system is as a Microsoft Access file format accdb which is the outcome of present research. By opening this file, the main page of this program named “Main form” will appear. This is the main page to access the program by a user according to the provided guidance. By choosing “entry of neonatal information”, a page titled “form of entering information” will be opened. The necessary information of the patient will be entered into database by cumminating with the system through the neonatal information form. This form contains demographics, clinical and administrational information fields about infant (Figure 2). Thus, some suitable forms have been designed for this purpose. These forms contain necessary requirements for entring, storing and retrieving and deleting and editing data. To design a form of entering information, data entering boxes, selecting check boxes and labels have been introduce

This page contains many different frames each of which contains adaptive objects with the type of the variables to be recorded. The left side of the page (A) involves the boxes for entering demographic and basic information of the baby obtained at the admission time. The doctor enters the clinical information of the baby into middle page (B); in this part different attributed icons will be appeared for entering the information concerning motherhood, reason of admissions, neonatal resuscitation, associated anomalies, blood culture, drug information, etc. The user enters the diagnostic codes corresponding to international system ICD-10 to the upper and middle parts of the frame (C) and the code of the cause of death can be entered also, if the baby dies. The user can add the baby’s photo on the top right part of the page (D) which is possible by double clicking on the frame of the photo followed by choosing and recording the baby’s photo. The lower part of the page (E) for entering neonatal discharge information. Then, the user chooses either the icon of death discharge or icon of discharge followed by entering the corresponding information. Again, there are some keys in the main data entering page for main operations (F). These keys are at the top right corner of the form devoted for storing, cancelling, adding, retrieving, etc. on the data.

### 3.3 System Implementation in a Hospital Setting and Displaying Data

The designed electronic system of summarized file has been implemented in the intensive care unit for newborns in Taleghani Hospital.

The forms of summarized record are set beside the patient soon after admission in clinical ward which will be filled by the doctor and nurses in different parts during the course of taking care. Then, the paper forms of information of newborn are collected to be given to the software weekly or monthly. Usually the summarized forms are then given to the doctor after being filled and completed by nurses for each discharged baby to be investigated and confirmed. Then after saving the information of the baby in the database, a copy of it will be kept in the ward and also one copy of it will be added to the file of the baby. The information being given to the software is actually kept in the table of baby’s database. At the moment, this system is going to be used in the NICU unit in hospitals Taleghani and 29 Bahman of Tabriz, and its database involves a massive raw data of newborn infants that are used to evaluate the performance and monitoring. The main application of this program is that the stored database can be analyzed easily by some statistical software such as Excel and SPSS. For this purpose, the data in the access database is transferred to Excel environment so that the data and defined variables are keept with the same format. This is done simply by some clicking to open the database first and the exporting them into Excel environment. Thus, in addition to change the data in Excel environment, we can use data mining for modeling and also discover the relationships of the variables. Moreover the present table can be transferred to SPSS software easily without losing or changing any data. In this way, we gain two major advantages: first, we do not need to define the variable in SPSS environment again; second, the data with defined variables are transformed to the database with the same format.

### 3.4 Software Evaluation

Questionnaire form of evaluation was given to 12 persons of the neonatal intensive care staff including doctors, nurses, technicians of medical records who worked with this system. Then after being filled out the questionnaire by users, and analyzing the data obtained, satisfaction rate of the users has been evaluated showing 85% satisfied in general. The satisfaction rate in detail was as follow:, in terms of software usefulness was 88.7%, in terms of being easily used was 82.2%, in terms of being easily learned was 90.4% and in terms of user satisfaction was 78.7%. The rate of individual job satisfaction was as follows: nurses were with highest satisfaction 87.7%, technicians of medical records were 85.7% and physicians were 83%.

## 4. Discussion

Minimum data set are usually designed by the needs of the wards and suggestion of the physicians so there is no standard and complete minimum data set in the neonatal intensive care. The variables in the different MDS are usually the same but some of the variables are preferred according to the need and its importance in clinical assessment. Also the required variables with the content of baby’s information corresponding to the needs and changes must be updated annually. Therefore our designed MDS has no particular advantage comparing to MDS in Vermont Oxford, Canadian Neonate Network and Schulman, but it has been designed only in accordance with the needs of Taleghani hospital NICU and the specialist’s suggestion of this ward. The database designed by Schulman for the NICU of New york hospital has several tables (7) for each part of the baby’s information and then they have been connected to each other by using one to one and one to some relations to create a centered tree form of data model, but there is not any tree model of data in our program. Actually this kind of relation has been removed from our program since we need only the data of the baby to be saved and thus the infant is defined as a relational bridge between the tables and all other entities related to the infant. All the necessary variables are defined in separate tables for each part of the wards that stores the babys information.

Vinty Paul and colleagues at the University of Kansas have developed a database for the NICU in 2006 (Vantipalli R, 2006). They used SQL software and Microsoft Visual Basic to design the program but we have used the Microsoft Access and the computer programing written by the same language to design the program. The reasons for choosing Access was easy accessibility of this software along with its applicability on different systems of hospitals and also the data can be transferred easily to Excel and SPSS software. Compared to Kansas University program, our program has an easier environment and more shorten. There is a special window for each selected icon in their program and thus a considerable parts of the screen will be unused but our program has just one check box option for the majority of clinical data that should be ticked. Also there are many pages in their program that the user should enter the data in contrast to our case that the user works with just two pages and all the required items are set in two pages. Although, there are more empty windows to add extra comments about the conditions of the patient in their program when it is necessary which is the main advantage of their program but the main advantage of our program is that the collected data can be transferred to the SPSS environment from the database in a unified form without any changes. Moreover except the data about identification or those related to date, all other data can be analyzed completely by this Software since the clinical data are often recorded numerically and the other text data are converted into code form. Most of the data of the patient in database are stored numerically and text data can be converted into the code form after they have been transferred to the Excel environment. For example, let code 1 stands for the field of “Female” and code 2 stands for “male” or in the field of the kind of discharge, let code 1 stands for the case “normal”, let code 2 stands for the case “satisfied” and let code 3 stands for the case of “transfer”. Thus, all the required data needed for investigating the observation are ready in the database and since most of the data are numerically available so they can be analyzed easily by the statistical Software such as SPSS.

It is possible to enter the data to the database digitally at the point of care on- line and for the doctor or as pen based form for nurse in our designed program but it is applicable in the hospitals under research at present since this technology is expensive in Iran and also it is necessary to teach the staffs how to work with it. It is suggested that the hospitals spend on buying the technology for the NICU ward as well as teach the staffs how to use computerized database. Also a specific person should be assigned for recording and analyzing the data in this section. Of course this person should be familiar with the medical concepts and the importance of health along with being familiar with computer science well enough to be able to handle the program efficiently.

**Limitations**

At first, the hardware and software is expensive in Iran and the second limitation is that the medical staffs are not well skilled in working with computer. Besidesely; low level of collaboration in Iran would be the third limitation.

## References

[ref1] Amani F., Barak M., Amini S. N., Dehghan Mohammad H. (2006). Neonatal Mortality and Its Related Factors in Hospitals of Ardabil 2002-2003. Journal Of Ardabil University Of Medical Sciences (JAUMS).

[ref2] Benbasat I., Goldstein D. K., Mead M. (1987). The case research strategy in studies of information systems. MIS quarterly.

[ref3] C, D. Microsoft Retrieved GUI Building MIT from http://ocw.mit.edu/index.htm.

[ref4] Cavaye A. L. (1996). Case study research: a multi - faceted research approach for IS. Information systems journal.

[ref5] Chin J. P., Diehl V. A., Norman K. L. (1988). Development of an instrument measuring user satisfaction of the human-computer interface. Paper presented at the Proceedings of the SIGCHI conference on Human factors in computing systems.

[ref6] Eddy D. M. (2005). Evidence-based medicine: A unified approach. Health affairs.

[ref7] Escobar G. J., Fischer A., Kremers R., Usatin M. S., Macedo A. M., Gardner M. N. (1997). Rapid retrieval of neonatal outcomes data: The Kaiser Permanente neonatal minimum data set. Quality Management in Healthcare.

[ref8] Fraser H. S., Biondich P., Moodley D., Choi S., Mamlin B. W., Szolovits P. (2005). Implementing electronic medical record systems in developing countries. Informatics in primary care.

[ref9] Herbst A., Drenth C. (2012). The intensity of intensive care: A patient's narrative. Global Journal of Health Science.

[ref10] Horbar J. D., Soll R. F., Edwards W. H. (2010). The Vermont Oxford Network: A community of practice. Clinics in perinatology.

[ref11] Kirk T., Levy A. Y. (1998). User interface for information retrieval system: Google Patents.

[ref12] Kliegman R., Behrman R. E., Jenson H. B., Stanton B. F. (2012). Nelson textbook of pediatrics: Elsevier/Saunders.

[ref13] Kohn L. T., Corrigan J. M., Donaldson M. S. (2000). To err is human: building a safer health system (Vol. 627). National Academies Press.

[ref14] Ntuli S. T., Malangu N., Alberts M. (2013). Causes of deaths in children under-five years old at a tertiary hospital in Limpopo province of South Africa. Glob J Health Sci.

[ref15] Orlikowski W. J., Baroudi J. J. (1991). Studying information technology in organizations: Research approaches and assumptions. Information Systems Research.

[ref16] Palvia P., Leary D., Mao E., Midha V., Pinjani P., Salam A. (2004). Research Methodologies In Mis: An Update. Communications of the Association for Information Systems, 14.

[ref17] Rachatapantanakorn O., Tongkumchum P., McNeil N. (2009). A Method on Assessing Complication-Base Risk Factors for Neonatal Morbidity: Application for Pattani Hospital Delivery. Global Journal of Health Science.

[ref18] Rahmanpour M. L. M., Afshar E. (2009). Evaluation of development of information and communication technologies in IRAN and Other Countries. Journal of Management Culture.

[ref19] Saeed M., Lieu C., Raber G., Mark R. (2002). MIMIC II: a massive temporal ICU patient database to support research in intelligent patient monitoring. Paper presented at the Computers in Cardiology, 2002.

[ref20] Schulman J. (2008). Managing Your Patients’ Data in the Neonatal and Pediatric ICU: An Introduction to Databases and Statistical Analysis. John Wiley & Sons.

[ref21] Sharifzadeh G. R., Namakin K., Mehrjoofard H. (2008). An Epidemiological Study on Infant Mortality and Factors Affecting it in Rural Areas of Birjand. Iran J Pediatr.

[ref22] Shneiderman B. (1982). The future of interactive systems and the emergence of direct manipulation. Behaviour & Information Technology.

[ref23] Shneiderman S. B., Plaisant C. (2005). Designing the user interface.

[ref24] Shortliffe E. H. (1999). The evolution of electronic medical records. Academic Medicine.

[ref25] Sprague A. E., Dunn S. I., Fell D. B., Harrold J., Walker M. C., Kelly S., Smith G. N. (2013). Measuring quality in maternal-newborn care: developing a clinical dashboard. J Obstet Gynaecol Can.

[ref26] Vantipalli R, W. J, Barlow S. M. (2006). NICU oromotor database. CNL Technical Research Report. University of Kansas.

[ref27] Yin R. K. (1998). The abridged version of case study research: Design and method.

